# Fibroblast Heterogeneity in and Its Implications for Plastic and Reconstructive Surgery: A Basic Science Review

**DOI:** 10.1097/GOX.0000000000002927

**Published:** 2020-06-23

**Authors:** Heather E. desJardins-Park, Malini S. Chinta, Deshka S. Foster, Mimi R. Borrelli, Abra H. Shen, Derrick C. Wan, Michael T. Longaker

**Affiliations:** From the * Hagey Laboratory for Pediatric Regenerative Medicine, Division of Plastic and Reconstructive Surgery, Department of Surgery, Stanford University School of Medicine, Stanford, Calif.; † Institute for Stem Cell Biology and Regenerative Medicine, Stanford University School of Medicine, Stanford, Calif.

## Abstract

Fibroblasts’ integral role in tissue development, maintenance, and disease represents a fast-growing field of basic science research. Although fibroblasts were long thought to be a homogeneous cell population, recent research has illuminated the unforeseen complexity of these cells, giving rise to the rapidly expanding research field of “fibroblast heterogeneity.” Fibroblasts play a critical role in states of tissue fibrosis such as skin scarring, which affects hundreds of millions of patients annually and causes severe aesthetic, developmental, and functional morbidity. Beyond scarring, major organ fibrosis is an enormous public health concern responsible for nearly half of all deaths in the United States. Because fibrosis is a conserved response to tissue damage in all organs, the study of fibroblasts throughout the body may help us to understand their role in the conditions most relevant to plastic and reconstructive surgery—for instance, skin scarring (eg, from burns, traumatic lacerations, or surgical incisions), “pathological” scarring (hypertrophic scars, keloids), and capsular contracture. Here, we present a basic science review of fibroblast heterogeneity in wound healing, cancer, organ fibrosis, and human dermal architecture. The field of fibroblast heterogeneity is young, and many of the insights discussed have yet to be translated clinically. However, plastic surgeons stand in a unique position to bridge these discoveries into clinical realities. We hope this information can spur readers to consider both what questions in plastic surgery can be studied from the lens of fibroblast heterogeneity, and how these preclinical insights can be translated to improving care of our patients.

## INTRODUCTION

Fibroblasts are the cells responsible for producing extracellular matrix (ECM), the scaffolding that surrounds cells throughout the body. Fibroblasts are a major component of the stroma, the body’s supportive connective tissue. These cells are indispensable in tissue development and homeostasis, playing an integral role in supporting other cell types and defining the architecture of tissues and organs.^1^ However, fibroblasts can also contribute substantially to disease.^2–4^ In particular, fibroblasts play a critical role in fibrosis, which can affect any organ in the body and lead to impaired function.^4^ Fibrosis is the final common pathway in many forms of tissue damage in both skin and viscera. States of fibrosis are defined by pathologic fibroblast activity, in which cells produce excessive amounts of abnormally organized ECM, leading to the replacement of functional native tissue with dense, nonfunctional connective tissue.^5^ Fibrosis causes an enormous burden of morbidity and mortality worldwide and is estimated to be responsible for 45% of all deaths in the United States.^4^ Skin scarring from surgery alone affects over 100 million patients per year in the developing world.^6^

Fibroblasts were historically thought to be a very primitive cell type. However, basic science research has progressively shown that fibroblasts are active in intercellular signaling and play a critical role in many developmental processes, physiologic functions, and pathologies.^7^ In particular, scientific interest in fibroblasts has grown rapidly in recent years due to work illuminating the concept of “fibroblast heterogeneity.”^2,8^ Although fibroblasts were long believed to be a homogeneous cell population, recent work has shown fibroblasts to be a strikingly diverse family of cells with wide-ranging functions throughout different anatomical sites, organs, physiologic processes, and disease states.^2,8–12^ The importance of fibroblasts in numerous processes central to the practice of plastic surgery—wound healing and scarring, skin development and maintenance, and cancer, among others—makes the expanding field of fibroblast heterogeneity of particular interest to our specialty.

The pace of research into fibroblasts and fibroses is accelerating, and although exciting developments have been made in recent years, much remains to be explored in the field of fibroblast heterogeneity. Although key aspects of fibroblast biology have begun to inform novel clinical directions in plastic surgery,^13–15^ most of the basic science insights that have defined the field of fibroblast heterogeneity have yet to be translated to clinical practice. However, plastic and reconstructive surgeons offer a unique firsthand understanding of soft-tissue biology and fibrosis. This places plastic and reconstructive surgeons in an ideal position to both advance the field of fibroblast biology and bridge the gap between preclinical research and novel clinical solutions.

This article aims to provide an overview of the current state of knowledge in fibroblast biology in a range of physiologic and disease states: wound healing, cancer, organ fibrosis, and human dermal physiology. Improved understanding of the different types of fibroblasts within the skin and other tissues could not only expand our understanding of fibrotic diseases and their underlying pathophysiologic mechanisms, but also yield novel insights into the treatment and prevention of fibrosis. Given that fibrosis is a conserved response to tissue damage throughout the body, insights into fibroblast heterogeneity in the diverse settings discussed here may inform potential therapeutic directions for treating those fibrotic conditions most relevant to plastic and reconstructive surgery. It is the authors’ hope that this review will provide our readers with a broad foundation to consider novel ways to leverage fibroblast heterogeneity for the benefit of plastic and reconstructive surgeons and our patients.

## FIBROBLASTS IN WOUND HEALING

**Fig. 1.**
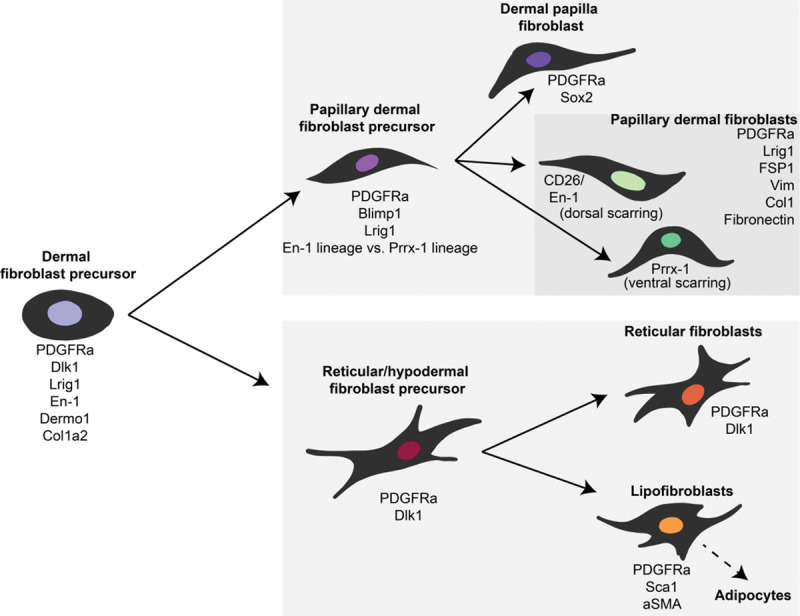
Dermal fibroblast heterogeneity. Hierarchy of murine dermal fibroblast subpopulations and their identifying molecular markers over the course of development and differentiation. Lineage/cell surface markers shown in this figure are based on data presented in the following publications: Driskell and Watt,^7^ Driskell et al,^12^ Borrelli et al 2020 (unpublished), and Rinkevich et al.^10^ PDGFRa, platelet-derived growth factor receptor alpha; Sox2, SRY-box transcription factor 2; Lrig1, leucine-rich repeats and immunoglobulin-like domains 1; FSP1, fibroblast-specific protein 1; Col1, collagen type 1; En-1, engrailed homeobox 1; Prrx-1, paired related homeobox 1; Dlk1, delta-like non-canonical notch ligand 1; Dermo1=Twist2, Twist-related protein 2; Col1a2, collagen type 1 alpha 2 chain; Sca1, stem cell antigen 1

Wound healing is one of the most well-researched examples of fibrosis in the body. As plastic surgeons are well aware, any injury involving the dermis—whether a burn, surgical incision, or other tissue trauma—will yield a fibrotic scar. Skin scars affect hundreds of millions of patients every year,^6^ resulting in an over $12 billion annual market for treatments.^16^ Fibroblasts, the cellular culprits of scarring, mediate ECM deposition in both dermal development and wound repair. Although the specific fibroblast subpopulations and intrinsic and extrinsic cues governing wound repair remain to be fully elucidated, several key discoveries in the field of dermal fibroblast heterogeneity have been made in recent years. An overview of mouse dermal fibroblast subsets and their defining surface markers is shown in Figure 1; the contributions of these cell populations to wound healing are discussed below.

A 2013 study by Driskell et al^12^ reported that unwounded mouse skin comprises 2 distinct fibroblast lineages defined by unique surface marker profiles: one that contributes to the papillary (superficial) dermis and one that forms the reticular (deep) dermis. The reticular lineage, which is characterized by active ECM production, is primarily responsible for dermal wound repair, potentially explaining the dense ECM-rich nature of scar tissue. In 2015, Rinkevich et al^10^ further defined the role of dermal fibroblast subpopulations in wound repair, reporting that a specific fibroblast lineage defined by *En1* expression and expression of the surface marker dipeptidyl peptidase-4 (Dpp4; also known as cluster of differentiation 26, CD26) is responsible for the vast majority of dorsal scarring in mice. Single-cell methods have also demonstrated striking heterogeneity among murine wound fibroblasts; one study found 12 separate clusters based on transcriptional profiles.^9^ The full diversity of fibroblast contributors to wound healing and scarring continues to be actively explored.

**Fig. 2.**
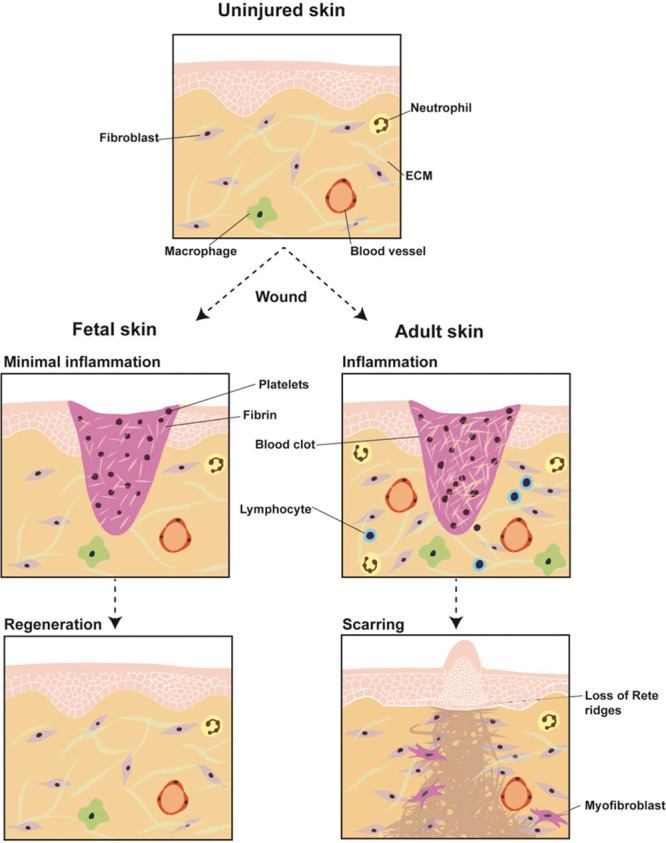
Scarless versus scarring healing. Early gestation fetal wound repair represents a paradigm for regenerative/scarless healing. Fetal skin (left) exhibits minimal inflammation following wounding (middle row left) and heals in a scarless fashion by regenerating normal skin; fetal fibroblasts produce ECM indistinguishable from that of unwounded skin (bottom row left). Relative to fetal skin, adult skin (right) exhibits markedly increased inflammation upon wounding (middle row right). In response to injury, postnatal fibroblasts produce scar tissue distinguished by dense, fibrotic ECM and a grossly raised appearance (bottom row right).

Further investigation of fibroblast contributions to scarring has been facilitated by comparing fibroblasts in different wound healing outcomes—ie, “normal” scarring versus decreased or increased fibrosis. For example, several studies have contrasted fibroblasts from the dermis (which heals via scarring) and oral mucosa (which is minimally scarring).^17^ In their 2015 article, Rinkevich et al^10^ demonstrated via reciprocal fibroblast transplantation that the fibroblasts responsible for tissue repair in the mouse oral mucosa (defined by *Wnt1* expression) are intrinsically nonfibrotic, whereas *En1*-positive fibroblasts from the dorsal dermis are intrinsically scar producing. This finding suggested that cell-intrinsic fibroblast differences may contribute to distinct healing outcomes between these sites. The profibrotic dermal fibroblast phenotype has also been correlated to increased CD26 expression compared with gingival fibroblasts in humans.^18^ Oral mucosal fibroblasts (which are neural crest-derived^10,19^) also demonstrate multipotential capacity^19^ and diminished propensity to differentiate into an activated myofibroblast phenotype in vitro,^20^ potentially explaining their increased regenerative capacity and decreased fibrosis. In addition, decreased myofibroblast contractility and responsiveness to mechanical stress (an important factor in wound fibroblast activation) have also been observed in fibroblasts derived from pig oral mucosa,^21^ *Acomys* mice,^22^ and mammalian fetal dermis,^23^ all of which represent examples of regenerative healing. An overview of regenerative, or “scarless,” healing is shown in Figure 2.

## FIBROBLASTS IN KELOID AND HYPERTROPHIC SCARS

On the other end of the spectrum, fibroblast differences have also been demonstrated to contribute to pathologic (hyperproliferative/hyperfibrotic) healing outcomes in the human skin. Fibroblasts from hypertrophic scars exhibit alterations in connective tissue deposition and related molecular signaling in response to wound molecular cues.^24,25^ Further, one study demonstrated phenotypic variation in fibroblasts from different dermal layers, and specifically implicated fibroblasts localized within the deep dermis in hypertrophic scarring.^26^ This finding is consistent with the observation in mice that the deeper dermal layers contain more profibrotic fibroblast subpopulations^12^ and suggests that in humans, like in mice, functional variation may exist between fibroblasts derived from different regions of the dermis.

Keloid-derived fibroblasts are functionally distinct from hypertrophic scar fibroblasts. Although these pathologies share key features (notably the excess connective tissue deposition characteristic of hyperproliferative scarring), keloids differ from both normal and hypertrophic scars in that they extend beyond the borders of the initial wound and never stop growing. Consistent with the fact that keloids grow continually and are in fact thought to be benign tumors, keloid-derived fibroblasts exhibit decreased apoptosis and *p53* expression, features commonly associated with tumors.^27^ Interestingly, mutations in the *p53* gene have also been identified in hypertrophic scar fibroblasts by one study; however, these mutations were rarer and less functionally significant, as phenotypically, hypertrophic scar fibroblasts did not exhibit the decreased rates of apoptosis seen in keloid fibroblasts.^28^ The distinctions between keloid and hypertrophic scar fibroblasts likely reflect the fact that these two scar outcomes are not merely different points on the same spectrum; rather, they have fundamentally different underlying pathophysiology, and as such may require distinct therapeutic approaches.

Interestingly, although fibroblasts are classically thought to be a completely differentiated cell type, recent research has shown that wound myofibroblasts may be less terminally differentiated and more “plastic” than previously believed. In 2017, Plikus et al^29^ reported that in murine wound healing, myofibroblasts are reprogrammed to become adipocytes by hair follicle–related signaling, a finding that was replicated in vitro in human keloid fibroblasts. The authors suggested that this transition of profibrotic fibroblasts into adipocytes may be a useful therapeutic target for reducing fibrosis in the setting of hyperproliferative scarring. Given the lack of effective treatment options for skin scarring and particularly for pathologic scarring, such as hypertrophic scars and keloids, a study of fibroblast heterogeneity across different healing outcomes may illuminate novel directions for therapeutic development.

## FIBROBLASTS IN CANCER

Similar to their function in dermal wound healing, fibroblasts comprising the stroma of solid tumors play an integral role in supporting tumor cell proliferation and regulating the tumor microenvironment.^3,30,31^ In fact, in a process known as the “serum response,” which is conserved between different tissue and tumor types, these cancer-associated fibroblasts (CAFs) recapitulate wound-healing gene expression pathways.^32^ However, as in wound healing and fibrosis, significant heterogeneity also exists between fibroblasts from different tumor types and sites as well as between species. As a result, despite their critical role in disease progression, fibroblasts are particularly challenging to target therapeutically in the setting of cancer.

Striking fibroblast heterogeneity can be seen in skin cancers. In melanoma, for example, fibroblasts expressing the cell surface marker CD26 are an important subpopulation of cells contributing to tumor stroma ECM deposition; in a mouse xenograft model of melanoma, depletion of the CD26-positive fibroblast subpopulation decreased tumor growth.^10^ In basal cell carcinoma, CAFs are known to express a variety of chemokines associated with both local immunosuppression and tumor progression. Interestingly, even fibroblasts in cancer-free, sun-damaged areas near patients’ tumors show cancer-associated gene expression patterns, suggesting that these cells might promote tumor formation.^33^

In breast carcinoma, 4 unique subpopulation of CAFs have been identified based on cell surface markers in humans.^34^ However, such delineation by cell surface markers alone is likely insufficient to capture the full extent of fibroblast heterogeneity because subtle changes in gene expression can yield distinct functional outcomes that may or may not be significantly reflected at the cell surface level.^35^ The complexity of CAFs may further be understood with regard to how these cells regulate other cell types, both cancer cells and immune cells, in the tumor microenvironment. For instance, melanoma cells co-cultured with fibroblasts exhibit decreased apoptosis in response to cisplatin, indicating that fibroblasts may support the development of melanoma cell drug resistance.^36^ CAFs in melanoma have also been shown in vitro to decrease natural killer (NK) cell-killing efficacy by their secretion of matrix metalloproteinases, suggesting an immune-modulatory role for CAFs.^37^

Expression of α smooth muscle actin (αSMA) is associated with subsets of “activated” (ie, profibrotic) fibroblasts in a variety of fibrotic pathologies and cancer types.^38–40^ Interestingly, in oral carcinoma, a subtype of CAFs with *low* αSMA expression was recently found to have an inhibitory effect on tumor proliferation and cancer cell self-renewal in vitro.^41^ Such results suggest a dual role for fibroblasts in tumors, with certain subpopulations encouraging tumor proliferation and other subpopulations involved in limiting it, both of which may be clinically targetable. Further investigation of CAF diversity within different tumor types is an active area of current research in the field. Given the increasing recognition of the important roles CAFs play in tumor development, progression, and treatment response, a deeper comprehension of these cells is imperative for identifying novel treatments and improving therapeutic management of cancer.

## PARENCHYMAL FIBROBLASTS AND ORGAN FIBROSES

**Fig. 3.**
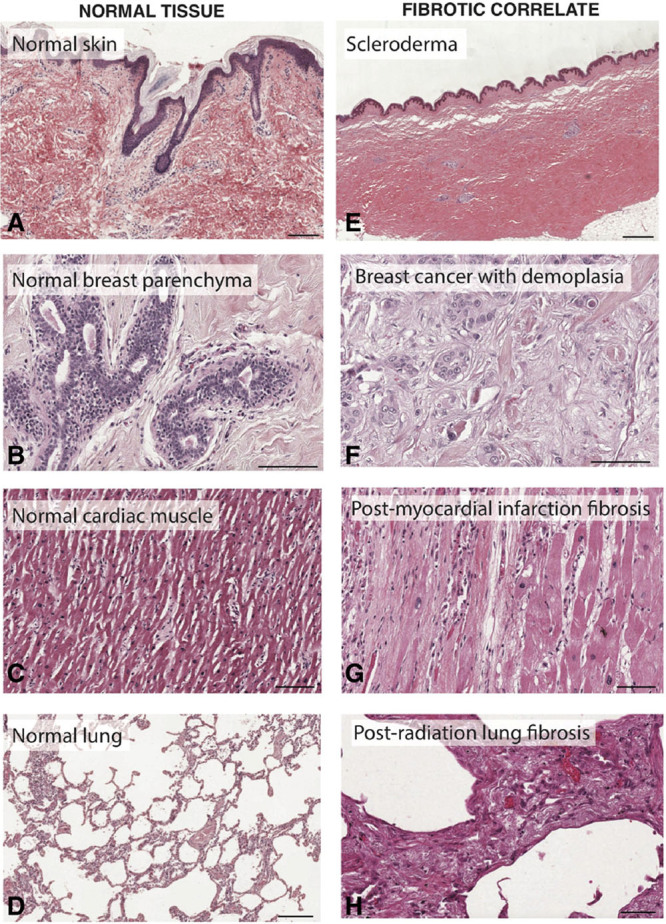
Organ fibrosis throughout the body. Representative histology of healthy tissue from the skin (A), breast (B), heart (C), and lung (D), compared with fibrotic tissue from those organs. Fibrotic tissue histology (E–H) demonstrates typical “hallmarks” of fibrosis, including densely aligned ECM fibers, decreased cellularity, and altered tissue architecture. Scale bars, 200 μm. Individual histology images were obtained from the Pathology Education Instructional Resource (PEIR) Digital Library and used with permission from Dr. Peter Anderson.

Distinct fibroblast subpopulations also exist within organ fibroses (Fig. 3), which often precede cancer. Studying fibroblast contributions in these organs may represent a paradigm from which we can gain insights into other fibroblast-driven pathologies.

In the lungs, mesenchymal myofibroblasts are known to be the primary cellular culprit of pulmonary fibrosis, but the subtypes and contributions of these cells remain poorly defined.^40,42^ Using single-cell RNA sequencing (scRNA-seq) of mouse lungs, Zepp et al^43^ revealed that alveolar niche cells mediate alveolar growth/regeneration, whereas distinct mesenchymal progenitor cells give rise to the myofibroblasts in pulmonary fibrosis. Xie et al^44^ further clustered mouse pulmonary mesenchymal cells into 7 distinct populations based on gene expression; these included myofibroblasts, lipofibroblasts, and multiple matrix fibroblast types, and *Pdgfrb*^*High*^ fibroblasts were specifically implicated in lung fibrogenesis.

In the liver, it is classically believed that during injury, hepatic stellate cells and portal fibroblasts transition to profibrotic myofibroblasts.^45^ One study suggested that mouse *Wt1*^+^ mesothelial cells differentiate into myofibroblasts during fibrogenesis, and that this transition can be prevented via transforming growth factor beta (TGFβ) antagonism.^46^ Great interest also exists regarding fibroblasts’ role in peritoneal adhesions, an extremely common postoperative sequela with a high readmission risk^47^ affecting over half of all abdominal/pelvic surgical patients.^48^ Tsai et al^49^ identified PDPN^+^MSLN^+^ (PDPN, podoplanin; MSLN, mesothelin) mesothelial cells as culprits of adhesion formation in a mouse surgical model. These cells upregulated hypoxia-inducible factor 1-alpha (HIF1α) expression; HIF1α inhibition significantly reduced adhesions.

The study of fibroblast heterogeneity may also yield insights into fibroses in other tissues commonly encountered in plastic surgery, such as the skin and breast. Capsular contracture is a fibrotic process frequently observed following a breast implant placement; although its mechanisms are poorly understood, the role of fibroblasts is an active topic of research.^50^ Studies have found that estrogen receptor expression by capsular myofibroblasts in patients is associated with an increased αSMA expression and capsular thickness,^51,52^ while clinically, antiestrogenic therapy is associated with a less-severe contracture,^52^ suggesting a potential therapeutic strategy for targeting capsular myofibroblasts.

Fibroblasts also play a critical role in chronic dermal fibroses, such as scleroderma (systemic sclerosis). However, although significant research has explored the molecular signaling governing both normal and pathologic dermal fibroblast behavior^53^—for example, it is evident that fibroblasts are regulated by TGFβ and Wnt signaling in states of fibrosis^54^—precise determinants of the fibroblast transition to a profibrotic phenotype, as well as the specific cell populations involved, remain to be elucidated. Further investigation into cellular culprits of fibrosis throughout the body, as well as interactions among different cell types (eg, fibroblasts and immune cells), may reveal conserved mechanisms relevant to treating diverse fibroses. Continued research may ultimately enable targeting of specific profibrotic fibroblast subpopulations to prevent/treat fibroses.

## HUMAN DERMAL FIBROBLAST DIVERSITY

**Fig. 4.**
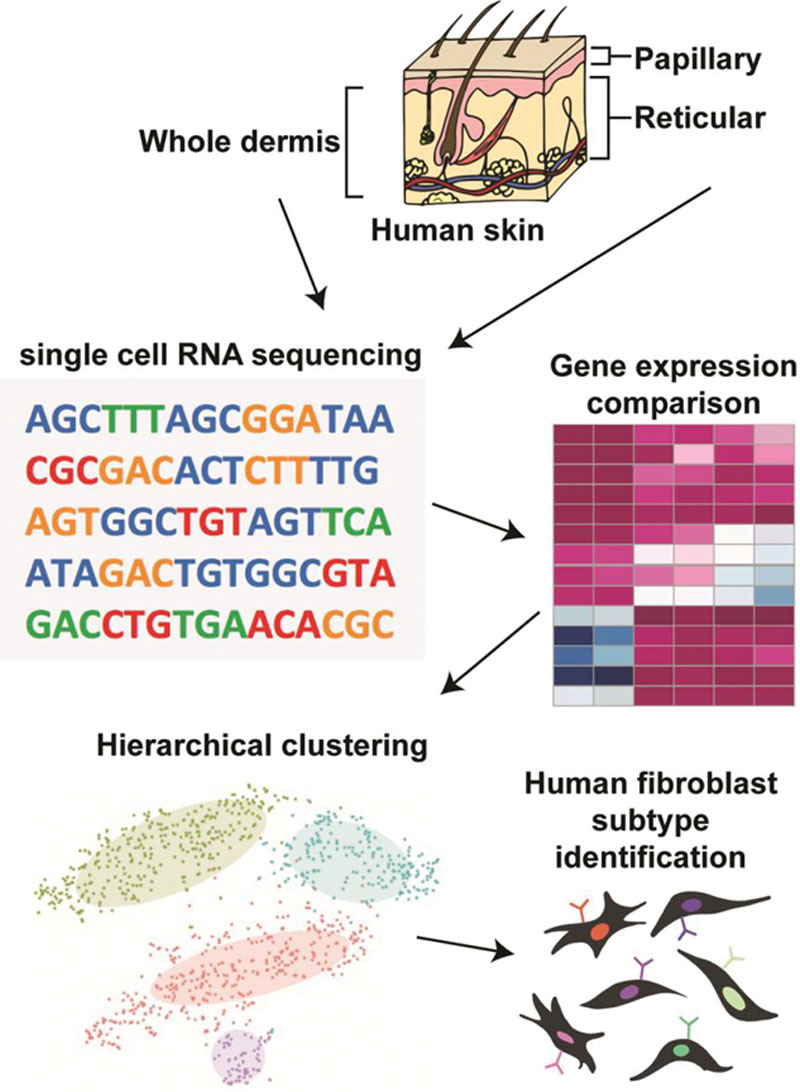
Defining human dermal fibroblast heterogeneity. The study of fibroblast heterogeneity in humans, as well as the identification of distinct dermal fibroblast subpopulations, has largely relied on single-cell molecular profiling. Generally, fibroblasts are isolated from either whole dermis or skin layers that have been anatomically separated (eg, using a dermatome) (A). These fibroblasts are then subjected to single-cell sequencing (B). Based on comparison of gene expression levels (C), fibroblasts can be clustered into subgroups that share similar transcriptional profiles (D), which may represent distinct fibroblast subpopulations (E).

The skin is the most well-researched example of fibroblast heterogeneity, and the most relevant to plastic and reconstructive surgery. To translate experimental work to novel regenerative medicine therapies, we must directly study fibroblast diversity in the human skin. Although fate-mapping experiments in mice are facilitated by transgenic animal models, such strategies cannot be translated to humans. In vitro methods have a limited utility, as cell culture significantly changes fibroblasts’ genetic signature.^55^ As such, the study of human dermal fibroblast subpopulations has relied on alternative strategies (Fig. 4).

One hypothesis-driven approach has been to exploit knowledge of murine fibroblast subpopulations to screen for analogous populations in the human skin. Shook et al^56^ identified a population of CD9^+^CD26^High^ myofibroblasts that were increased in mouse wounds and were also present in the human skin. However, key differences exist between mouse and human skin physiology and wound healing.^57,58^ These differences may be reflected in divergent surface markers, as observed in other tissues.^59,60^ For example, while Sca-1 expression has been used to distinguish fibroblast subsets in mice,^2^ no human ortholog of this surface marker exists.^61^

Another popular approach to exploring human fibroblast heterogeneity has been interrogation of fibroblast subpopulations by spatial segregation within the dermis. The mammalian dermis is divided into the papillary (superficial) dermis (which is highly cellular) and the reticular (deep) dermis (which is rich in collagen and connective tissue).^62^ In mice, these regions harbor distinct fibroblast subpopulations.^63,64^ In humans, gene expression analyses revealed a higher expression of immune- and angiogenesis-related genes in papillary fibroblasts, and a higher expression of genes associated with cytoskeleton organization and connective tissue formation in reticular fibroblasts.^65,66^ Korosec et al^67^ identified 2 cell surface markers distinguishing papillary and reticular fibroblasts: papillary fibroblasts (FAP^+^CD90^−^  - FAP, fibroblast activation protein; CD90, cluster of differentiation 90) expressed PDPN, NTN1 (netrin 1), and higher CD26 levels (consistent with findings in mice^10,12,63^), whereas reticular fibroblasts (CD90^+^) expressed ACTA2 (actin alpha 2, smooth muscle; also known as α-SMA, alpha smooth muscle actin), MGP (matrix Gla protein), PPARy (peroxisome proliferator-activated receptor gamma), and CD36. Interestingly, papillary fibroblasts had increased proliferative potential but could not give rise to adipocytes, whereas CD90^+^CD36^+^ reticular fibroblasts readily underwent adipogenic differentiation.^68,69^ It should be noted that these distinctions are not comprehensive; for example, 10% of reticular dermal cells express CD36, whereas a subset of papillary fibroblasts express the endothelial marker CD146, suggesting a perivascular fibroblast subpopulation. Further, it is possible that “intermediate” layers between the reticular and papillary dermis harbor additional fibroblast lineages.^70,71^

Single-cell sequencing promises an unbiased approach for studying human fibroblast heterogeneity. Philippeos et al^72^ performed scRNA-seq on CD90^+^ and CD90^−^ cells (96 each) isolated from abdominal skin of a single donor. Hierarchical clustering defined 4 fibroblast subpopulations—1 upper dermal, 1 lower dermal, and 2 reticular dermal clusters—with distinct gene expression profiles. Tabib et al^73^ conducted scRNA-seq on full-thickness forearm skin biopsies from 4 patients. Gene expression hierarchical clustering of fibroblasts (*COL1A1*^*+*^) identified 2 major subpopulations (expressing *SFRP2/DPP4* and *FMO1/LSP1*, respectively) and 5 minor subpopulations. However, the fact that these markers identified by RNA-seq are largely intracellular impedes prospective cell isolation and complicates comparisons to work describing fibroblast subpopulations only by surface markers (eg, Philippeos et al^72^).

The initial findings concerning fibroblast heterogeneity in the human skin are promising. However, many areas of further exploration remain uninitiated, including whether these transcriptionally heterogeneous fibroblast subpopulations represent different cellular states or truly distinct cell lineages. A critical direction of research will be how these fibroblast subsets change in different physiologic and disease states, such as aging.^74^ As our understanding of human dermal fibroblasts progresses, these findings will be critical in informing treatment for a broad range of skin conditions and pathologies.

## DISCUSSION

Fibroblasts are a diverse collection of cells that are integral for tissue homeostasis and maintenance as well as for response to damage (eg, wound healing).^75^ Key discoveries have been made in recent years with regard to different fibroblast subtypes, particularly within the skin, and their contributions to both physiologic and pathologic processes. For example, as discussed earlier, recent studies have identified distinct profibrotic and proregenerative fibroblast subtypes within different tissues, highlighting fibroblasts’ functional diversity.^17^ These discoveries have the potential to inform novel therapeutic directions; for instance, basic science discoveries of mechanical signaling pathways driving profibrotic fibroblast behavior have been translated into therapies to reduce scarring by targeting wound tension.^13–15^ Additional insights into fibroblast heterogeneity, signaling, and lineage hierarchies may allow researchers to target specific fibroblast populations and cell signaling pathways to prevent fibrosis in the dermis and other tissues, with the potential to expand therapeutic strategies available to plastic and reconstructive surgeons.

**Fig. 5.**
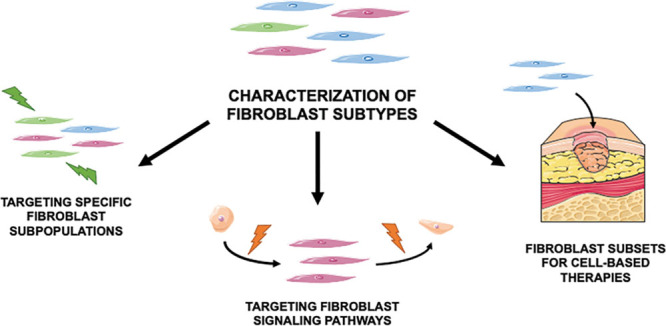
Therapeutic directions for fibroblast heterogeneity. Although basic science research in fibroblast heterogeneity has rapidly uncovered new knowledge about different fibroblast subtypes (top) and their functions, most of this knowledge remains to be clinically translated. We envision several possible broad directions for translating from the bench to the bedside. Bottom left: identification of fibroblast subsets involved in specific pathologies, such as scarring and fibroses, may enable those specific cells to be targeted to prevent and treat disease. Bottom middle: understanding of the precise signaling pathways mediated by fibroblast subpopulations and governing their behavior may reveal novel molecular targets to manipulate fibroblast behavior clinically. Bottom right: with the growing importance of cell- and tissue engineering–based therapeutic approaches, identification of the specific functions of different fibroblast types (eg, profibrotic vs. proregenerative) may enable particular fibroblasts with desired functions to be applied directly for treatment, such as in chronic wounds. Figure includes elements obtained from Servier Medical Art (http://smart.servier.com), licensed under a Creative Common Attribution 3.0 Generic License.

However, important limitations remain with regard to our knowledge of fibroblast heterogeneity. Lack of consensus regarding specific subpopulations, lineage restriction, and cell signaling among fibroblasts has a complicated definition of their precise roles in fibrotic pathophysiology. Additionally, given the fact that fibroblasts’ interactions with other cell types (eg, keratinocytes) influence their biology,^76^ it will be critical to characterize fibroblasts’ intercellular signaling and roles within their in vivo niche. Finally, and most importantly, much of our knowledge of fibroblast heterogeneity has yet to be translated into clinical solutions that improve patient care. Plastic and reconstructive surgeons have an unparalleled ability to guide this translation. As practitioners of a uniquely innovative and creative medical specialty, plastic surgeons are no strangers to the interface where cutting-edge preclinical research and novel clinical solutions meet. In addition, plastic and reconstructive surgeons have an intimate firsthand familiarity with the biology of soft tissue and the macroscopic bodily processes that are fundamentally driven by fibroblasts. The value of such surgical intuition should not be overlooked: plastic surgeons are ideally positioned to develop creative applications of these basic science discoveries to surgical practice. We envision several broad lenses through which the knowledge of fibroblast heterogeneity may be used to inform novel treatments; these are illustrated in Figure 5.

## CONCLUSIONS

Herein we have reviewed the wide-ranging functionality of fibroblasts in numerous physiologic and pathologic processes such as dermal wound healing, cancer, and internal organ fibrosis. Although many questions remain unanswered regarding the cellular identity of fibroblast subpopulations and the mechanisms governing their behavior, advancements made in the study of fibroblast heterogeneity have already revealed valuable insights into fibrosis. Although fibroblasts in other tissues remain less well-explored compared with those in the dermis, the principles that dictate dermal fibroblast heterogeneity and pathologic contributions may yield knowledge applicable to other tissue types, and vice versa. Investigation of the cellular basis of fibrotic disease represents a step toward developing novel treatment regimens for combating fibrosis not only in the skin, but throughout the body. We hope that this article can inspire our readers to consider what unanswered questions remain in fibroblast heterogeneity, and how preclinical research in this field can be adapted to drive innovative clinical solutions in plastic and reconstructive surgery.
